# Why We Are Right Handed

**Published:** 1889-09

**Authors:** 


### WHY WE ARE RIGHT-HANDED.

Primitive man, being by nature a fighting animal, fought for the most part at first with his great canine teeth, his nails and his fists, till in process of time he added to those early and natural weapons the further persuasions of a club or shillalah. He also fought, as Darwin has conclusively shown, in the main for the possession of the ladies of his kind, against other members of his own sex and species. And if you fight you soon learn to protect the most exposed and vulnerable portion of your body. Or, if you don't, natural selection manages it for you, by killing you off as an immediate consequence.

To the boxer, wrestler, or hand-to-hand combatant, that most vulnerable portion is undoubtedly the heart. A hard blow, well delivered on the left breast, will easily kill, or at any rate, stun even a strong man. Hence, from an early period men have used the right hand to fight with, and have employed the left arm chiefly to cover the heart and to parry a blow aimed at that specially vulnerable region. And when weapons of offense and defense supercede mere fists and teeth, it is the right hand that grasps the spear or sword, while the left holds over the heart for defense, the shield or buckler.

From this simple origin then, the whole vast difference of right and left in civilized life takes its beginning. At first, no doubt the superiority of the right hand was only felt in the manner of fighting. But that alone gave it a distinct pull, and paved the way at last for the supremacy elsewhere. For when weapons came into use, the habitual employment of the right hand to grasp the spear, sword or knife, made the nerves or muscles of the right side far more obedient to the control of the will, than those of the left. The dexterity thus acquired by the right—see how the word "dexterity" implies this fact—made it more natural for the early hunter and artificer to employ the same hand preferentially in the manufacture of flint hatchets, bows and arrows, and all the other manifold activities of savage life. It was the hand with which he grasped his weapon; it was therefore the hand with which he chipped it. To the end, however, the right hand remains especially "the hand in which you hold your knife;" and that is exactly how your own children to this day decide the question which is which, when they begin to know their right hand from their left for practical purposes.

